# 16S rRNA, metagenomics and 2bRAD-M sequencing to decode human thanatomicrobiome

**DOI:** 10.1038/s41597-024-03518-3

**Published:** 2024-07-06

**Authors:** Xin Huang, Jianye Zeng, Shilin Li, Ji Chen, Hongyan Wang, Chengtao Li, Suhua Zhang

**Affiliations:** 1 Institute of Forensic Science, Fudan University, Shanghai, 200032 PR China; 2 The State Key Laboratory of Genetic Engineering and MOE Key Laboratory of Contemporary Anthropology, School of Life Sciences, Fudan University, Shanghai, P.R. China; 3 Department of Forensic Medicine, School of Basic Medical Sciences, Fudan University, Shanghai, 200032 PR China

**Keywords:** Microbiology, Microbial communities

## Abstract

Microorganisms are essential in the decomposition of corpses and play a significant role in forensic science. However, previous studies have primarily focused on animal remains, specifically the gut, skin, and burial environment. Insufficient research has been conducted on the microbiota of human cadavers, especially in cases of advanced decomposition and additional tissues, resulting in a lack of relevant reference data. In this study, the microbiota of eight cadavers at different stages of decomposition were detected using 16S rRNA, metagenomic sequencing and 2bRAD-M sequencing. Nine different sites, including oral and nasal cavities, heart, liver, spleen, lung, kidney, muscle and gut, were analysed and the efficacy of these methods was evaluated. The results showed that 16S rRNA sequencing was the most cost-effective method for the study of cadavers in the early stages of decomposition, whereas for cadaveric tissues in the late stages of decomposition, 2bRAD-M could overcome host contamination more effectively than metagenomic sequencing. This paves the way for new opportunities in data retrieval and promotes in-depth investigations into the microbiota.

## Background & Summary

The Human Microbiome Project (HMP) has linked variations in microbiota structure to human health, demonstrating the integral role of microorganisms in the human body, and the Integrative Human Microbiome Project (iHMP) has elucidated the interaction between microorganisms and the host^1^.Currently, there is extensive research and application of the distribution and function of human microbial communities during life. However, there is limited knowledge of post-mortem microbial changes. It has been demonstrated that microbial succession continues beyond individual death, and hosts’ death can be viewed as an ecological disruption to the microbiota^2^. Microorganisms play a crucial role in carcass decomposition^3^, but more research is needed to determine how microorganisms change during decomposition and correlate with environment, season, host, *et al*. Therefore, exploring the structure and changes of the thanatomicrobiome and elucidating the time-dependent change profiles of them during the stages of autolysis, putrefaction and decomposition of cadavers is crucial for forensic research.

The development and maturity of DNA sequencing technology drive the interpretation of microbiota structure and diversity in different environments. Presently, Amplicon sequencing^4^ and Metagenomic Sequencing^5^ are the most mainstream DNA sequencing technologies for microbiome research. Amplicon sequencing overwhelmingly targets the 16S rRNA gene (bacteria and archaea)^6^, and the internal transcribed spacer (ITS) region (fungi)^7^. 16S rRNA sequencing is fast, easy and can be applied to large-scale studies, thus has now become the most commonly used technique for cadaver microbiological research. However, this technique offers low strain resolution which only reaches the genus level, and many of the annotations do not extend to the species level^8^. Metagenomic sequencing does not require targeted amplification in advance, and the sample DNA is directly segmented randomly for library sequencing. The entire genetic material in the microbiome sample is sequenced^9^. This technique allows species identification at species level^10^ with high accuracy. Nevertheless, this technique is high-cost and still challenging to be applied to samples with high levels of host nucleic acid background^11^. 2bRAD-M is a recently-emerged innovative technology, and it is developed based on 2b-RAD technology. This technique uses type IIB restriction enzymes to digest the genome to produce an equal length enzyme tag. Subsequently, the fragments are enriched and amplified by ligating them with adaptors, and libraries are then constructed for sequencing. The obtained sequencing results are later compared with the unique 2bRAD tag database (2b-Tag-DB) for qualitative and relative quantitative analysis^12^. Previous study reported that 2bRAD-M was able to detect microbes with highest accuracy and sensitivity, and analyze samples with low biomass (down to 1 pg), severe degradation, or high contamination^13^. At present, there is no report on the application of thanatomicrobiome detection to this technology. Among these three different methods, which one is more suitable for cadaver microorganism research remains unclear.

**Fig. 1:**
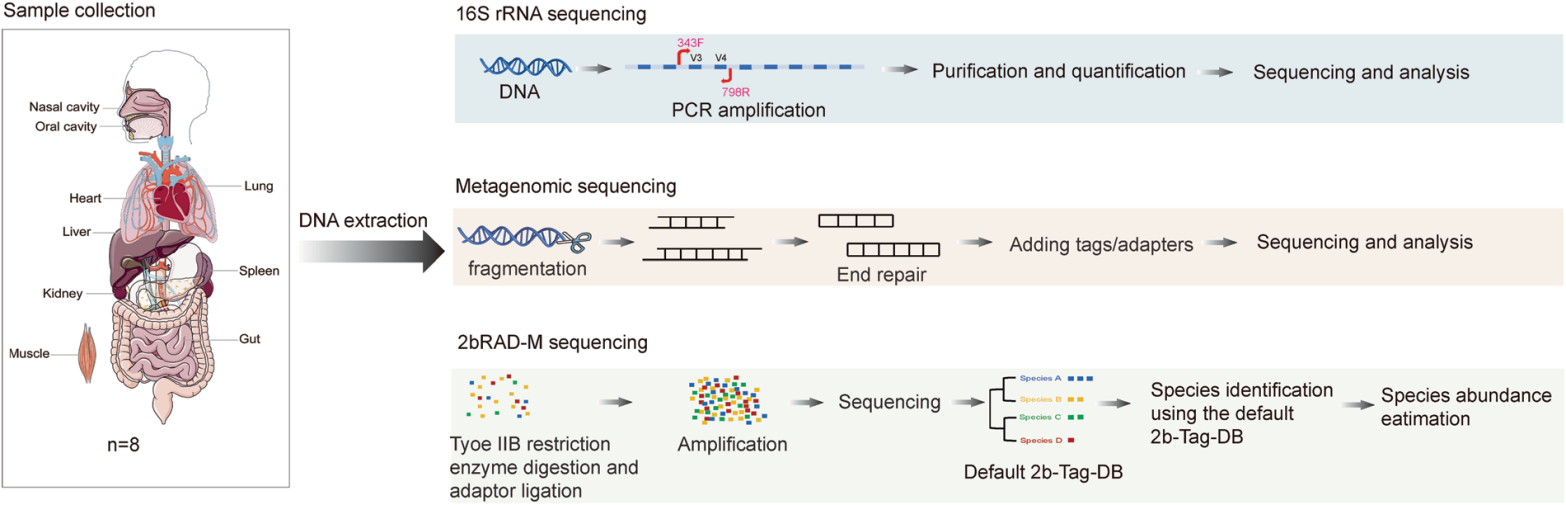
A schematic overview of the study workflow.

In this study, the microbiota in nine distinct body regions (including oral and nasal cavities, hearts, livers, spleens, lungs, kidneys, muscles and guts) of eight cadavers at different stages of decomposition were examined using the three aforementioned methods—16S rRNA, metagenomic sequencing, and 2bRAD-M. The relevant details of the samples can be found in Table 1. Two of the bodies were autopsied within 24 hours, whereas body S03 was autopsied 71 days post mortem and showed the most advanced decomposition. Table 2 displays the extracted DNA quality report. Multiple DNA tissue samples from individual S03 were inadequate for metagenomic sequencing. A schematic overview of the study workflow is shown in Fig. 1.

**Table 1:** Sample information of eight cadavers in this study.

Individual ID	Age	Gender	PMI (days)	Cause of Death	Sample state
S01	54	Male	9	Craniocerebral injury	Frozen
S02	64	Male	15	Sudden cardiac death	Frozen
S03	69	Female	71	Severe anemia	Unfrozen (limb corpse green)
S04	87	Female	22	Coronary heart disease	Frozen
S05	80	Male	5	Stomach tumor and lung infection	Unfrozen (Thoracic abdominal corpse green)
S06	41	Male	1	Craniocerebral injury	Unfrozen
S07	65	Female	22	Coronary heart disease	Frozen
S08	47	Male	1	Coronary heart disease	Unfrozen

**Table 2:** Extracted DNA quality report of samples.

Tissue type	Individual ID	Sample ID	DNA quality rate*	Tissue type	Individual ID	Sample ID	DNA quality rate*
Oral cavity	S01	S1-1	A	Spleen	S04	S5-4	A
	S02	S1-2	A		S05	S5-5	A
	S03	S1-3	C		S06	S5-6	A
	S04	S1-4	A		S07	S5-7	C
	S05	S1-5	A		S08	S5-8	A
	S06	S1-6	A	Lung	S01	S6-1	A
	S07	S1-7	A		S02	S6-2	A
	S08	S1-8	A		S03	S6-3	C
Nasal cavity	S01	S2-1	A		S04	S6-4	A
	S02	S2-2	A		S05	S6-5	A
	S03	S2-3	A		S06	S6-6	A
	S04	S2-4	A		S07	S6-7	A
	S05	S2-5	A		S08	S6-8	A
	S06	S2-6	A	Kidney	S01	S7-1	A
	S07	S2-7	A		S02	S7-2	C
	S08	S2-8	A		S03	S7-3	C
Heart	S01	S3-1	A		S04	S7-4	C
	S02	S3-2	A		S05	S7-5	A
	S03	S3-3	A		S06	S7-6	A
	S04	S3-4	A		S07	S7-7	C
	S05	S3-5	A		S08	S7-8	A
	S06	S3-6	A	Muscle	S01	S8-1	A
	S07	S3-7	A		S02	S8-2	A
	S08	S3-8	A		S03	S8-3	A
Liver	S01	S4-1	A		S04	S8-4	A
	S02	S4-2	A		S05	S8-5	A
	S03	S4-3	C		S06	S8-6	A
	S04	S4-4	A		S07	S8-7	A
	S05	S4-5	A		S08	S8-8	A
	S06	S4-6	A	Gut	S03	S9-3	C
	S07	S4-7	A		S04	S9-4	A
	S08	S4-8	A		S05	S9-5	A
Spleen	S01	S5-1	A		S06	S9-6	A
	S02	S5-2	A		S07	S9-7	A
	S03	S5-3	C		S08	S9-8	C

*Criteria for DNA quality determination: (A) Quality meets test requirements (C) Incomplete quality meets experimental requirements, which may lead to library construction failure.

Quality control data (Supplementary Figure 1) indicates a range of 58,215 to 74,123 clean reads for 16S rRNA sequencing, with valid tags after chimera removal varying from 42,609 to 73,551. The oral and nasal cavity samples displayed higher microbial diversity compared to other body regions. Metagenomic sequencing showed substantial host contamination, leading to significant data loss.

**Fig. 2:**
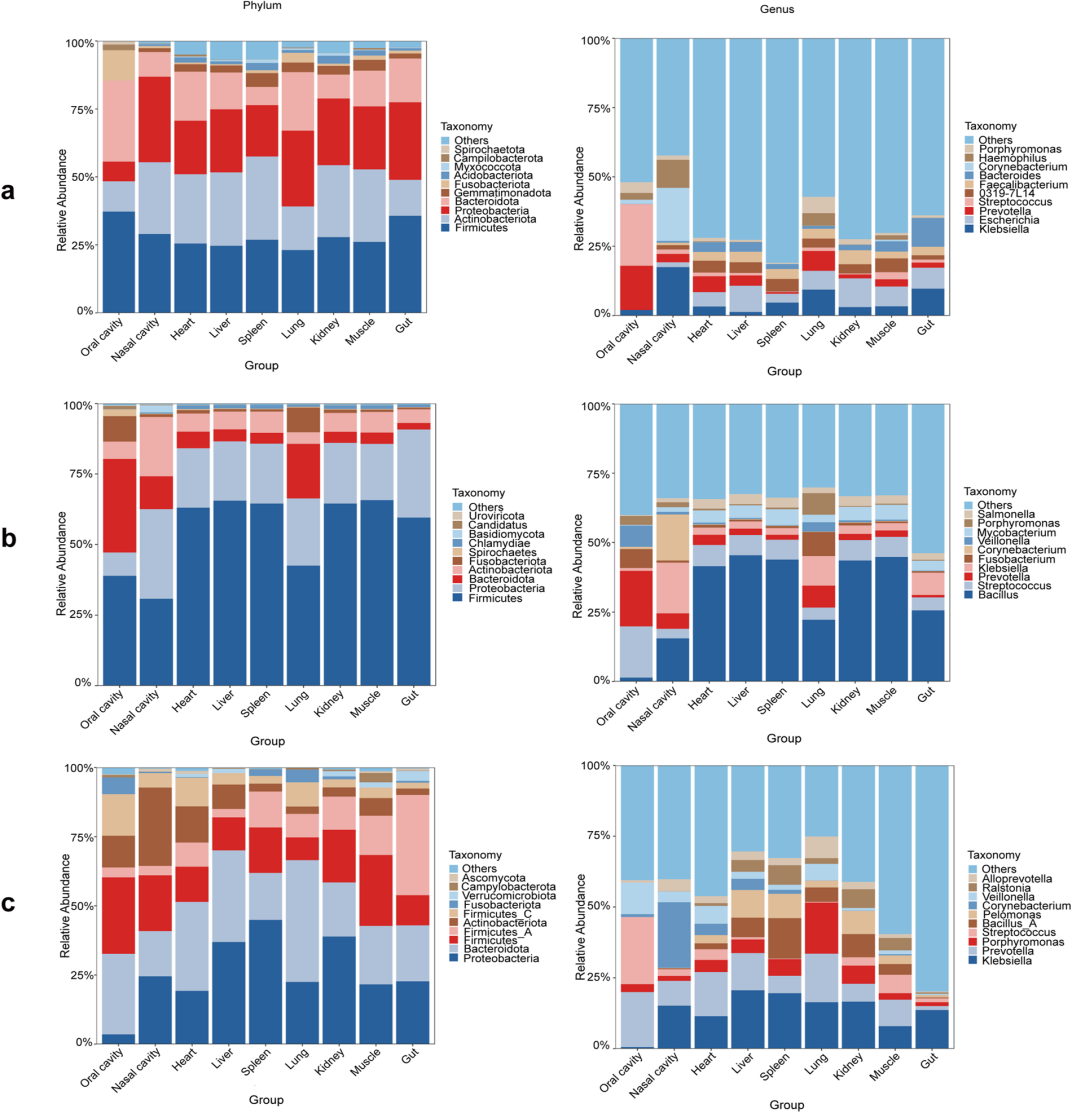
10 Most abundant members of the microbial community and their relative abundances at phylum and genus level. (**a**) Results of 16S RNA analysis (**b**) Results of metagenomic analysis (**c**) Results of 2bRAD-M analysis.

Community structure analysis revealed that the thanatomicrobiome primarily clustered into five phyla: *Bacteroidetes*, *Firmicutes*, *Actinobacteria*, *Fusobacteria* and *Proteobacteria*, with *Firmicutes* being the most predominant across all sample groups, corroborated by both 16S RNA and metagenomic analyses (Fig. 2). Significant differences were observed in the species composition of the oral cavity group compared to others, with *Bacteroidota* and *Firmicutes* being the most prevalent. The 2bRAD-M data showed distinct dissimilarities, especially with the presence of *Proteobacteria* varying in abundance across different groups. At the genus level, the 16S RNA sequencing highlighted poor resolution, with many species categorized as “others.” However, metagenomic analysis identified core microbiotas in the oral cavity, such as *Streptococcus* and *Prevotella*, which was consistent with previous studies. In contrast, the nasal cavity group showed a prevalence of *Bacillus* and *Klebsiella*, with 2bRAD-M revealing a widespread presence of *Klebsiella* across various groups.

**Fig. 3:**
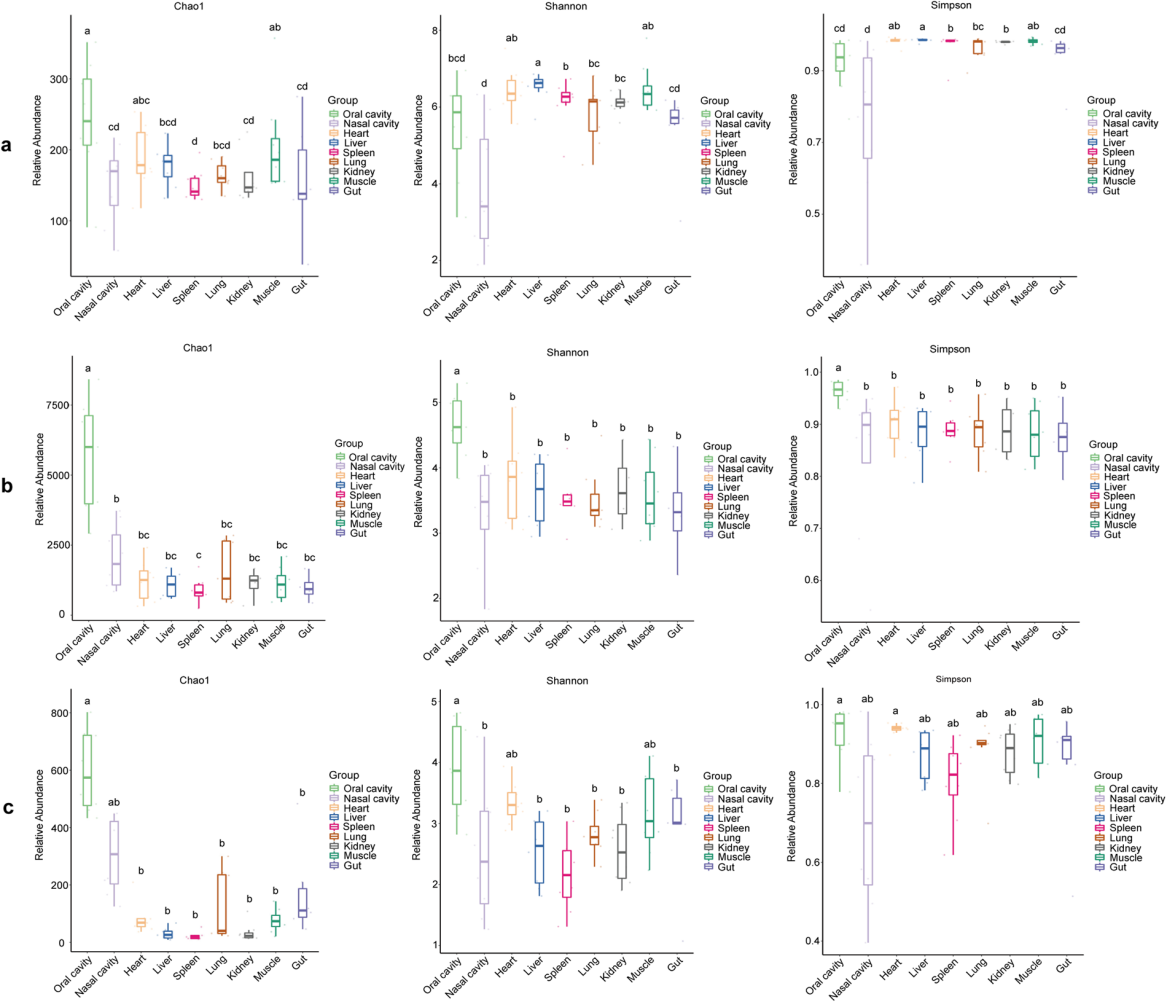
Alpha diversity of the microbial community in each group. (**a**) Results of 16S RNA analysis (**b**) Results of metagenomic analysis (**c**) Results of 2bRAD-M analysis. Same letter denoted above two groups indicates there is no significant difference between them. Conversely, significant differences between them do exist.

**Fig. 4:**
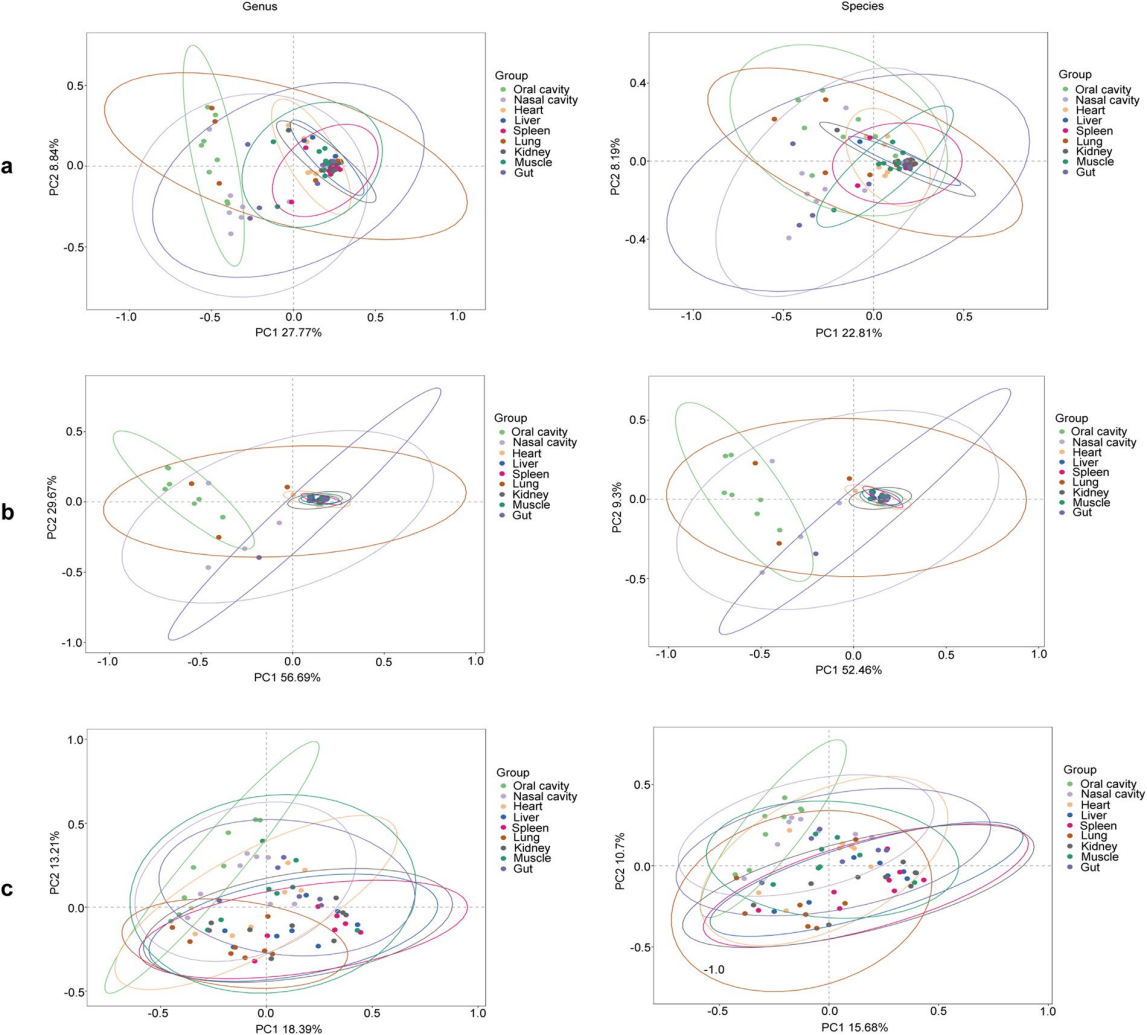
Beta diversity of the microbial community in each group at genus and species levels (**a**) Results of 16S RNA analysis (**b**) Results of metagenomic analysis (**c**) Results of 2bRAD-M analysis.

Alpha diversity metrics like Chao1, Shannon, and Simpson indexes were used to evaluate species diversity and evenness within the groups. The oral group exhibited the highest microbial diversity, particularly noted in the Chao1 index values, while the nasal cavity group had notably lower species evenness (Fig. 3). Beta diversity analysis through PCoA at both genus and species levels showed that samples from the same group generally clustered together, although the lung samples displayed more dispersion (Fig. 4).

**Fig. 5:**
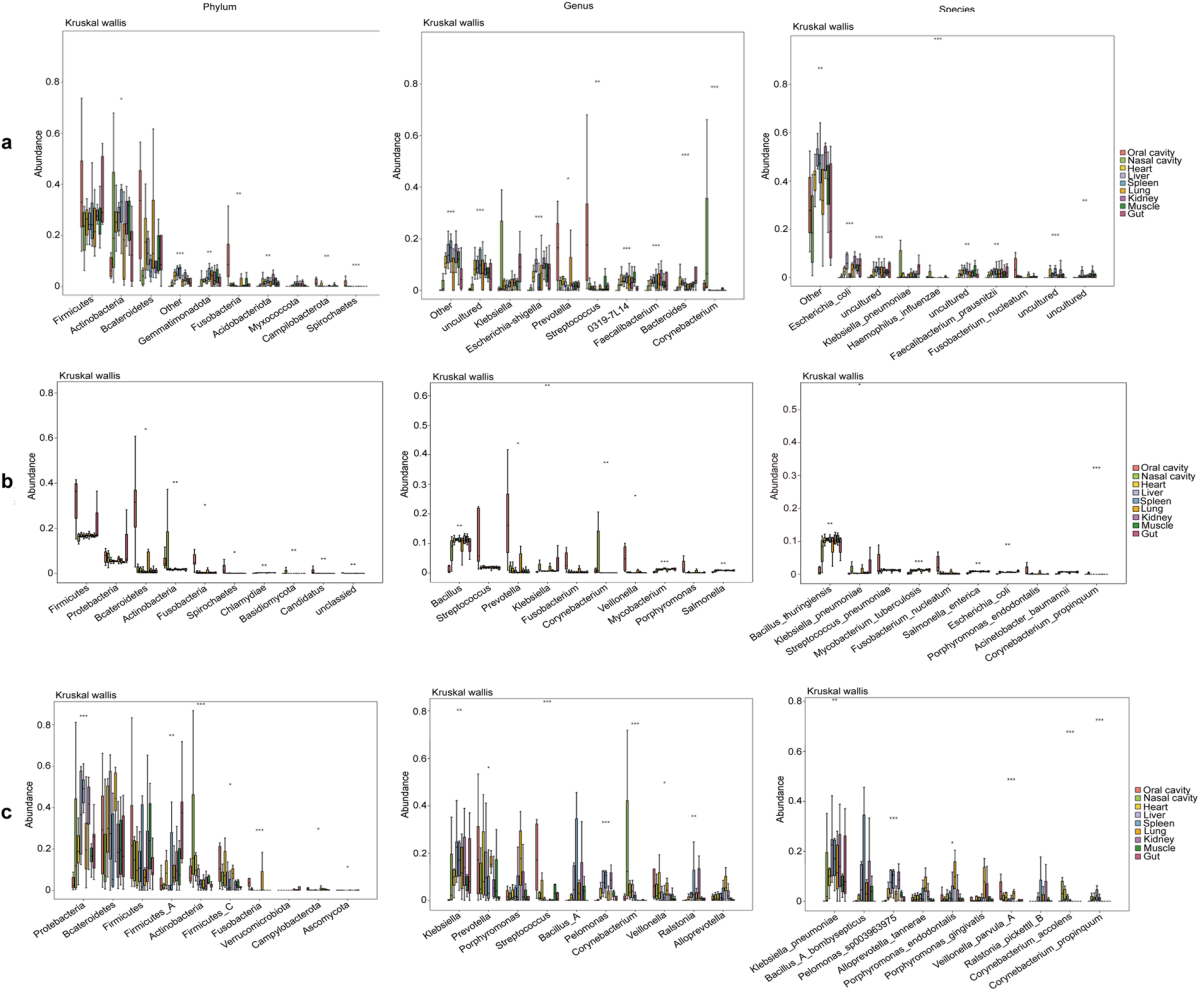
Boxplot of abundances of the 10 species with greatest significant differences. (**a**) Results of 16S RNA analysis (**b**) Results of metagenomic analysis (**c**) Results of 2bRAD-M analysis.

Differences in species abundance were assessed using Kruskal-Wallis analysis, which highlighted *Firmicutes* and *Bacteroidetes* as consistently differing phyla across all techniques. At the genus level, the 16S RNA analysis suggested a low resolution with significant detections being categorized under “other” and “uncultured.” In contrast, metagenomic and 2bRAD-M analyses identified *Streptococcus*, *Prevotella*, and *Klebsiella* as key differing genera. Notably, *Klebsiella pneumoniae* emerged as the most divergent species among the groups (Fig. 5).

The findings underscore the varying capabilities of the three sequencing techniques, with each showing specific strengths in identifying and analyzing microbial communities in cadaveric samples. While 16S rRNA sequencing offers rapid insights, its lower resolution at the species level limits its depth of analysis. Metagenomic sequencing, although more comprehensive, is challenged by host contamination. The 2bRAD-M method shows promise for high-accuracy analysis in degraded samples, but requires further database expansion. These insights could guide the selection of appropriate sequencing techniques in future forensic microbiological research, aiming for accurate postmortem interval estimations and broader applications in microbial forensics.

## Methods

### Ethical statement

This study involving the use of cadaver tissues was conducted in strict accordance with the ethical standards of the Fudan University and with the 1964 Helsinki declaration and its later amendments. Prior to commencement of the study, approval was obtained from the Fudan University Ethics Committee (Approval Number: 20230301-014).

Informed consent was obtained in this study. For these deceased subjects which were subject to judicial autopsy, next of kin consent was obtained for the use of their biological samples in this research. All personal identifiers were removed from the samples prior to analysis to maintain confidentiality and comply with data protection regulations. The handling of human tissues was performed by trained professionals and in compliance with the institutional safety protocols and the guidelines of Fudan University.

### Sample collection and DNA extraction

To ensure the sterility and integrity of human samples collected for microbiological analysis, a rigorous aseptic technique was employed^14,15^, and in accordance with the latest CDC guidelines for handling biological samples. Sample collection was performed in a controlled environment using sterilized equipment. Technicians conducting the collection wore sterile gloves, masks, and protective clothing to prevent contamination from the environment or personnel.

During the dissection process, the skin or surface of the tissue was first disinfected using 70% isopropyl alcohol and allowed to dry completely to minimize the risk of external microbial contamination. Using a sterile lancet, an incision was made, and the biological samples was collected directly into sterile, DNA/RNA-free containers, which were immediately capped to prevent airborne contamination. For the collection of swabs from oral and nasal cavity samples, we used sterile cotton swabs. All tools and containers were pre-sterilized using autoclaving at 121 °C for 15 minutes, ensuring the elimination of potential microbial presence as per the standards outlined by the American Society for Microbiology (ASM). The samples were transported to the laboratory under refrigerated conditions to prevent the growth of any incidental microbial contaminants and processed within one hours of collection to preserve the microbial integrity of the samples.

Total DNA was extracted from the samples using QIAamp^®^ Fast DNA Stool Mini Kit (Qiagen, Hilden, Germany). The DNA concentration and integrity were assessed by NanoDrop2000 spectrophotometer (Thermo Fisher Scientific, Waltham, MA, USA) and agarose gel electrophoresis, respectively.

### 16S RNA sequencing and data analysis

The 16S RNA sequencing used extracted DNA as the template for PCR amplification of bacterial 16S rRNA genes, along with the barcoded primers and Takara Ex Taq (Takara). The V3-V4 variable regions of 16S rRNA genes were amplified with universal primers 343 F (5′-TACGGRAGGCAGCAG-3′) and 798 R (5′-AGGGTATCTAATCCT-3′)^16^. PCR amplified products were visualized using agarose gel electrophoresis. Following electrophoresis, PCR products were purified using AMPure XP beads (Agencourt, USA) with a double-pass protocol to ensure high purity. The final purified amplicons were quantified using the Qubit dsDNA Assay Kit (Thermo Fisher Scientific, USA). The DNA concentrations were adjusted for high-throughput sequencing. Sequencing was performed on Illumina NovaSeq 6000 platform, generating 250-bp paired-end reads. The sequencing service was provided by OE Biotech Company (Shanghai, China). The raw data were acquired in FASTQ format.

Paired-end reads were initially processed with Cutadapt software to remove adapter sequences. Quality filtering and chimera removal were conducted using the DADA2 plugin in QIIME2 (v 1.9.1). Subsequently, the processed reads were assigned to amplicon sequence variants (ASVs) and quantified. Representative sequences for each ASV were aligned and annotated against the Silva database (v 138) using the q2-feature-classifier plugin with default parameters.

Diversity within samples (alpha diversity) was assessed using Chao1^17^, Shannon^18^, and Simpson indices, calculated in QIIME2. Beta diversity, indicating inter-sample microbial composition variation, was analyzed through Principal Coordinates Analysis (PCoA) using the unweighted UniFrac distance matrix. Statistical differences in microbial communities between groups were evaluated using the Kruskal-Wallis test, with a significance threshold of *P < *0.05, conducted in R.

### Metagenomic sequencing and data analysis

Total DNA was fragmented using S220 Focused-ultrasonicators (Covaris, USA). The fragmented DNA was then purified using AMPure XP beads. Libraries were constructed according to the manufacturer’s instructions using the TruSeq Nano DNA LT Sample Preparation Kit (Illumina, San Diego, CA, USA). Metagenomic sequencing was performed by OE Biotech Co., Ltd. (Shanghai, China) using an Illumina NovaSeq 6000 sequencing platform, which produced 150 bp paired-end reads. The raw sequencing output (FastQ files) underwent adapter removal using Trimmomatic (v 0.36) and quality filtering to eliminate low-quality bases. Host sequences were removed using Bowtie2 (v 2.2.9).

Valid reads were assembled into metagenomes using MEGAHIT (v 1.1.2)^19^. The assembled scaffolds were processed for open reading frame (ORF) prediction with Prodigal (v 2.6.3)^20^, and predicted ORFs were translated into amino acid sequences. CDHIT (version 4.7.0) was used to construct non-redundant gene sets from these sequences with clustering parameters set at 95% identity and 90% coverage. The longest gene from each cluster was selected as the representative sequence. Clean reads from each sample were aligned to the non-redundant gene set using Bowtie2 (v 2.2.9) to quantify gene abundances. Species annotations were assigned based on the taxonomy information corresponding to the NR library. Species abundances were calculated using the gene abundances linked to each species.

Alpha diversity indices including Chao1, Shannon, and Simpson were calculated using the vegan package in R (v 3.2.0). Beta diversity was assessed by computing Euclidean distances from species abundance data and visualized through Principal Coordinates Analysis (PCoA). Differences between groups were analyzed using the Kruskal-Wallis statistical test, consistent with the methods used for 16S RNA sequencing analysis.

### 2bRAD-M sequencing and data analysis

DNA ranging from 1 pg to 200 ng was digested with 4 U of BcgI enzyme (NEB) at 37 °C for 3 hours. Adaptors were then ligated to the digested DNA fragments in a reaction that combined 5 µl of digested DNA with 10 µl of ligation master mix. This mix included 0.2 µM of each adaptor and 800 U of T4 DNA ligase (NEB). The ligation was conducted at 4 °C over 12 hours. Following ligation, the products were amplified via PCR and resolved on an 8% polyacrylamide gel. DNA bands approximately 100 bp in size were excised and the DNA was subsequently eluted into nuclease-free water at 4 °C for 12 hours. Sample-specific barcodes were incorporated using PCR with platform-specific barcode-bearing primers. The PCR products were then purified using the QIAquick PCR Purification Kit (Qiagen) and prepared for sequencing. Sequencing was performed on the Illumina NovaSeq PE150 platform by OE Biotech Co., Ltd. (Qingdao, China). This process utilized the 2bRAD-M protocol.

A total of 173,165 microbial genomes, including bacteria, fungi, and archaea, were downloaded from the NCBI RefSeq database. Using a type IIB restriction endonuclease, restriction fragments were generated from these genomes to establish a comprehensive 2bRAD microbial genome database. Each set of 2bRAD tags sampled from the genomes was cataloged under its respective GCF number along with corresponding taxonomic information. Unique 2bRAD tags, occurring once within each genome, were identified and compared across the microbial genomes. These unique tags were developed into species-specific 2bRAD markers, contributing to the construction of a 2bRAD marker database.

Species composition, along with alpha and beta diversity analyses, were performed using methods consistent with those applied in 16S rRNA sequencing analysis. These included calculations of diversity indices and comparative analyses using the Kruskal-Wallis test to determine statistically significant differences between microbial communities.

## Data Records

All raw sequencing data generated during this study have been deposited in the NCBI Sequence Read Archive (SRA). The data records for this study include:

1. 16S rRNA Sequencing Data: The raw sequence data files (FastQ format) from the 16S rRNA sequencing are accessible under the BioProject ID: PRJNA1119244^21^.
2. Metagenomic Sequencing Data: The raw sequence data files (FastQ format) from the metagenomic sequencing are available under the BioProject ID: PRJNA1121222^22^.
3. 2bRAD-M Sequencing Data: The raw sequence data files (FastQ format) from the 2bRAD-M sequencing are stored in the SRA under the BioProject ID: PRJNA1120598^23^.

Additionally, comprehensive statistics related to the species abundance from the 16S rRNA, Metagenomic, and 2bRAD-M sequencing data are available on Figshare with the 10.6084/m9.figshare.24798567^24^. The specific sequences and procedures used to generate the data records are detailed in their respective subsections, linking directly to the corresponding data records.

## Technical Validation

Samples were taken aseptically by using sterilized equipment and sterile RNase and DNase-free tubes. To assess the potential for contamination, we also sampled the tissue surfaces separately from the internal tissues. DNA were extracted in an RNase free environment. DNA concentration and integrity were measured with NanoDrop 2000 (Thermo Fisher Scientific, USA) and agarose gel electrophoresis. For 16S rRNA gene amplification, negative control containing PCR-grade water and PCR with bacterial primers 343 F and 798 R were used. No positive or negative sequencing controls were used to obtain metagenomic data. The concentration of the 16S rRNA gene amplicons and controls was measured with a Qubit 2.0 Fluorometer and their quality were analysed using agarose gel electrophoresis.

The quality of the raw 16S rRNA sequencing reads was assessed using FastQC, which provided initial insights into potential issues such as poor quality scores, overrepresented sequences, or adapter contamination. Representative sequences for each ASV were aligned and annotated against the Silva database (v 138) using the q2-feature-classifier plugin with default parameters, ensuring reliable species-level identification. Metagenomic Sequencing Reads were subjected to stringent quality filtering steps using Trimmomatic, where bases below a quality score threshold of 20 were trimmed, and reads shorter than 75 bp after trimming were discarded. The sequencing depth and coverage were assessed to ensure that the metagenomic data were sufficient to capture a comprehensive snapshot of the microbial diversity present in the samples. Adequate coverage ensures that low-abundance species are represented. Potential contamination from host or other exogenous DNA was monitored and quantified using Kraken2, which aligns reads against a comprehensive database of known microbial and non-microbial sequences. The consistency and reproducibility of 2bRAD tag generation were verified by replicating the digestion and ligation reactions. This step confirmed that the enzyme cuts and adapter ligations were consistent across samples, crucial for comparative analyses. The alignment of 2bRAD tags to the reference microbial genome database was validated by checking the alignment scores and coverage. High alignment scores indicate accurate and reliable matching of tags to genomic sequences. The specificity and sensitivity of the developed species-specific 2bRAD markers were assessed by comparing the marker sequences against a panel of reference genomes. This ensured that markers were uniquely identifying the correct species without cross-reactivity.

Data from the different sequencing approaches were integrated to provide a holistic view of the microbial communities. Cross-validation among the datasets was conducted to ensure consistency and reliability across different sequencing platforms and methodologies.

### Supplementary information

Supplementary Information

## Data Availability

In this analysis, default parameters or parameters recommended by the developer were used.

## References

[1] Human Microbiome Project Consortium (2012). Structure, function and diversity of the healthy human microbiome. Nature.

[2] Martino C (2022). Microbiota succession throughout life from the cradle to the grave. Nat Rev Microbiol.

[3] Javan GT (2019). An interdisciplinary review of the thanatomicrobiome in human decomposition. Forensic Sci Med Pathol.

[4] Kolbert CP, Persing DH (1999). Ribosomal DNA sequencing as a tool for identification of bacterial pathogens. Current Opinion in Microbiology.

[5] Quince, C., Walker, A. W., Simpson, J. T., Loman, N. J., Segata, N. Shotgun metagenomics, from sampling to analysis. *Nature biotechnology* **35** (2017). 10.1038/nbt.3935 28898207

[6] Rj, C. *et al*. Use of 16S rRNA and rpoB genes as molecular markers for microbial ecology studies. *Applied and environmental microbiology* **73** (2007). 10.1128/AEM.01177-06 PMC1797146 17071787

[7] Cl, S. *et al*. Nuclear ribosomal internal transcribed spacer (ITS) region as a universal DNA barcode marker for Fungi. *Proceedings of the National Academy of Sciences of the United States of America* **109** (2012). 10.1073/pnas.1117018109 PMC3341068 22454494

[8] Ranjan R, Rani A, Metwally A, McGee HS, Perkins DL (2016). Analysis of the microbiome: Advantages of whole genome shotgun versus 16S amplicon sequencing. Biochem Biophys Res Commun.

[9] Quince C, Walker AW, Simpson JT, Loman NJ, Segata N (2017). Shotgun metagenomics, from sampling to analysis. Nat Biotechnol.

[10] Laudadio I (2018). Quantitative Assessment of Shotgun Metagenomics and 16S rDNA Amplicon Sequencing in the Study of Human Gut Microbiome. OMICS.

[11] Shi Y, Wang G, Lau HC-H, Yu J (2022). Metagenomic Sequencing for Microbial DNA in Human Samples: Emerging Technological Advances. Int J Mol Sci.

[12] Wang, S., Meyer, E, McKay, J. K, Matz, M. V. 2b-RAD: a simple and flexible method for genome-wide genotyping. *Nature methods* **9** (2012). 10.1038/nmeth.2023 22609625

[13] Sun Z (2022). Species-resolved sequencing of low-biomass or degraded microbiomes using 2bRAD-M. Genome Biol.

[14] de Jongh DS, Loftis JW, Green GS, Shively JA, Minckler TM (1968). Postmortem Bacteriology: A Practical Method for Routine Use. American Journal of Clinical Pathology.

[15] Fernández-Rodríguez A (2015). How to optimise the yield of forensic and clinical post-mortem microbiology with an adequate sampling: a proposal for standardisation. Eur J Clin Microbiol Infect Dis.

[16] Cw, N. *et al*. Design of 16S rRNA gene primers for 454 pyrosequencing of the human foregut microbiome. *World journal of gastroenterology* **16** (2010). 10.3748/wjg.v16.i33.4135 PMC2932916 20806429

[17] Chao A, Bunge J. Estimating the number of species in a stochastic abundance model. *Biometrics* **58** (2002). 10.1111/j.0006-341x.2002.00531.x 12229987

[18] Hill, T. C. J., Walsh, K. A., Harris, J. A. & Moffett, B. F. Using ecological diversity measures with bacterial communities. *FEMS microbiology ecology* **43** (2003). 10.1111/j.1574-6941.2003.tb01040.x 19719691

[19] Li D, Liu C-M, Luo R, Sadakane K, Lam T-W (2015). MEGAHIT: an ultra-fast single-node solution for large and complex metagenomics assembly via succinct de Bruijn graph. Bioinformatics.

[20] Hyatt D (2010). Prodigal: prokaryotic gene recognition and translation initiation site identification. BMC Bioinformatics.

[21] (2024). NCBI Sequence Read Archive.

[22] (2024). NCBI Sequence Read Archive.

[23] (2024). NCBI Sequence Read Archive.

[24] Huang X (2023). figshare.

