# Supplementary material for: 16S rRNA, metagenomics and 2bRAD-M sequencing to decode human thanatomicrobiome
Source: Sci Data. 2024 Jul 6;11:736. doi: 10.1038/s41597-024-03518-3 (PMC11227556; doi:10.1038/s41597-024-03518-3)
Supplement: Supplementary file 1 — Supplementary Information [file 41597_2024_3518_MOESM1_ESM.pdf]

Content

Figure S1 1

a

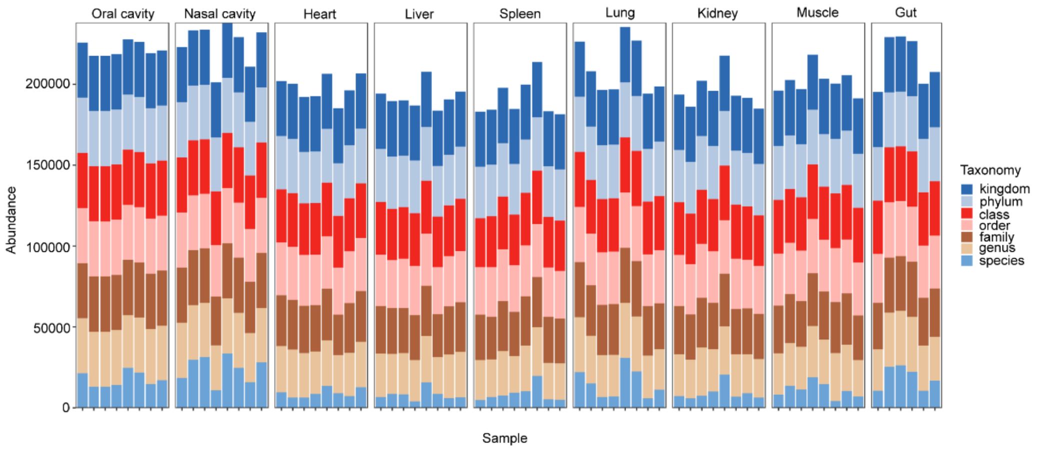

b

Group	Sample	total_reads	dehost_reads	percent
Oral cavity	S1-1	99647138	72622950	72.88%
	S1-2	97994898	3660896	3.74%
	S1-4	92556508	4167752	4.50%
	S1-5	99789924	25433014	25.49%
	S1-6	102682496	57718922	56.21%
	S1-7	98444456	5864658	5.96%
	S1-8	88867898	18834106	21.19%
Nasal cavity	S2-1	116955074	4561548	3.90%
	S2-2	110867710	1341256	1.21%
	S2-3	121997012	1430232	1.17%
	S2-4	117581734	1252292	1.07%
	S2-5	94213012	2874278	3.05%
	S2-6	88148342	13530978	15.35%
	S2-7	98132472	859562	0.88%
	S2-8	100278326	1489620	1.49%
Heart	S3-1	110537566	1364854	1.23%
	S3-2	100903484	954992	0.95%
	S3-3	113869566	1181044	1.04%
	S3-4	104247544	1064510	1.02%
	S3-5	102546254	1430246	1.39%
	S3-6	96204304	1010626	1.05%
	S3-7	105811390	1054894	1.00%
	S3-8	97057904	1121400	1.16%
Liver	S4-1	104521946	1458954	1.40%
	S4-2	101265858	1291486	1.28%
	S4-4	98033328	1080804	1.10%
	S4-5	105272108	1398726	1.33%
	S4-6	98326630	1096526	1.12%
	S4-7	85025058	1082602	1.27%
	S4-8	85750660	978580	1.14%

Group	Sample	total_reads	dehost_reads	percent
Spleen	S5-1	88016314	942036	1.07%
	S5-2	86833986	831524	0.96%
	S5-4	111448798	1024934	0.92%
	S5-5	94950502	964164	1.02%
	S5-6	104170766	1093146	1.05%
	S5-7	102628578	1149562	1.12%
Lung	S6-1	96780936	1298722	1.34%
	S6-2	93130122	970516	1.04%
	S6-4	105996248	1007464	0.95%
	S6-5	106754672	4549624	4.26%
	S6-6	104546798	2100914	2.01%
	S6-7	109468682	973044	0.89%
	S6-8	102344356	1003010	0.98%
Kidney	S7-1	99101090	1236920	1.25%
	S7-5	99955200	1085512	1.09%
	S7-6	91880448	955064	1.04%
	S7-8	100603504	996358	0.99%
Muscle	S8-1	118287554	1270852	1.07%
	S8-2	161024352	1280836	0.80%
	S8-3	152560654	1112002	0.73%
	S8-4	148134302	1082198	0.73%
	S8-5	152797178	1296062	0.85%
	S8-6	137981250	1038098	0.75%
	S8-7	155095752	1089744	0.70%
	S8-8	143358850	1118246	0.78%
Gut	S9-4	144092690	1196408	0.83%
	S9-5	119443566	1072792	0.90%
	S9-6	108314896	1984338	1.83%
	S9-7	109922650	811866	0.74%

Figure S1 Comparative analysis of microbial diversity and sequencing efficacy across different samples using 16S RNA and metagenomic sequencing techniques. (a) Numbers of ASVs in each sample at different taxonomic levels of 16S RNA sequencing. (b) Statistics of metagenomic sequencing reads.
